# Human Dental pulp stem cells (hDPSCs): isolation, enrichment and comparative differentiation of two sub-populations

**DOI:** 10.1186/s12861-015-0065-x

**Published:** 2015-03-14

**Authors:** Alessandra Pisciotta, Gianluca Carnevale, Simona Meloni, Massimo Riccio, Sara De Biasi, Lara Gibellini, Adriano Ferrari, Giacomo Bruzzesi, Anto De Pol

**Affiliations:** Department of Surgical, Medical, Dental and Morphological Sciences with interest in Transplant, Oncology and Regenerative Medicine, University of Modena and Reggio Emilia, Modena, Italy; Department of Biomedical, Metabolic and Neuroscience, University of Modena and Reggio Emilia, Children Rehabilitation Special Unit, IRCCS Arcispedale Santa Maria Nuova, Reggio Emilia, Italy

**Keywords:** DPSC, CD34, Neural crest, Neuromesenchyme, Multipotency

## Abstract

**Background:**

Human dental pulp represents a suitable alternative source of stem cells for the purpose of cell-based therapies in regenerative medicine, because it is relatively easy to obtain it, using low invasive procedures. This study characterized and compared two subpopulations of adult stem cells derived from human dental pulp (hDPSCs). Human DPSCs, formerly immune-selected for STRO-1 and c-Kit, were separated for negativity and positivity to CD34 expression respectively, and evaluated for cell proliferation, stemness maintenance, cell senescence and multipotency.

**Results:**

The STRO-1^+^/c-Kit^+^/CD34^+^ hDPSCs showed a slower proliferation, gradual loss of stemness, early cell senescence and apoptosis, compared to STRO-1^+^/c-Kit^+^/CD34^−^ hDPSCs. Both the subpopulations demonstrated similar abilities to differentiate towards mesoderm lineages, whereas a significant difference was observed after the neurogenic induction, with a greater commitment of STRO-1^+^/c-Kit^+^/CD34^+^ hDPSCs. Moreover, undifferentiated STRO-1^+^/c-Kit^+^/CD34^−^ hDPSCs did not show any expression of CD271 and nestin, typical neural markers, while STRO-1^+^/c-Kit^+^/CD34^+^ hDPSCs expressed both.

**Conclusions:**

These results suggest that STRO-1^+^/c-Kit^+^/CD34^−^ hDPSCs and STRO-1^+^/c-Kit^+^/CD34^+^ hDPSCs might represent two distinct stem cell populations, with different properties. These results trigger further analyses to deeply investigate the hypothesis that more than a single stem cell population resides within the dental pulp, to better define the flexibility of application of hDPSCs in regenerative medicine.

## Background

The stem cell field represents an area of particular interest for scientific research. New therapeutic strategies have been made possible, thanks to great advancements in stem cell biology, with the aim to regenerate tissues damaged by injuries or diseases [1,2]. Based on their ability to rescue and/or repair injured tissue and partially restore organ function, several types of stem/progenitor cells have been investigated. Stem cells can be described as undifferentiated cells that are characterized by three fundamental abilities: proliferation, self-renewal, and differentiation towards multiple cell lineages [3]. The differentiation process can be recognized by a change in cell morphology and by the expression of tissue-specific proteins [4].

Adult stem cells have been identified in many organs and tissues, including brain, bone marrow, peripheral blood, blood vessels, skeletal muscle, skin, teeth, heart, gut, liver, ovarian epithelium, and testis [5].

Among these tissues, dental pulp, a soft connective tissue contained within the pulp chamber of the tooth, is considered an interesting source of adult stem cells due to the high content of cells and to the low-invasive procedures required for cell isolation, compared to other adult tissue sources [6-8]. The first type of dental stem cell was isolated from the human pulp tissue and termed “postnatal dental pulp stem cells” (DPSCs) which are obtained from human permanent teeth [9]. Further types of dental-MSC-like populations were isolated and characterized: stem cells from human exfoliated deciduous teeth (SHEDs) [10], stem cells from apical papilla of human immature permanent teeth (SCAPs) [11-13]. Moreover, DPSCs can be also isolated from supernumerary teeth, which are generally discarded [13]. Other sources of dental stem cells are the periodontal ligament, which retains periodontal ligament stem cells (PDLSCs) [14] and the dental follicle, containing dental follicle progenitor cells (DFPCs) [15,16]. The typical surface markers of mesenchymal stem cells are CD44, CD73, CD90, CD105, CD271 and STRO-1, while the negative markers are CD34, CD45, and HLA-DR [17]. However there is no specific, strict marker characterizing DPSCs, which are considered a heterogeneous population.

Indeed, different mesenchymal stem cell markers were used to select different subsets of DPSCs displaying different biological behaviours [18]. STRO-1 which is considered a specific mesenchymal stem cells (MSC) marker, in DPSCs was also demonstrated to identify a subgroup of cells with odontogenic and osteogenic properties [19]. Another isolated DPSCs population revealed to be positive for CD34 and CD117 and negative for CD45 [20]. Particularly, c-Kit, a membrane tyrosin-kinase receptor, which specifically interacts with the stem cell factor (SCF), is expressed by different cell types. Its expression is well identified in melanocytes [21], hematopoietic stem cells [22], adipose stem cells [23] and bone marrow stem cells [24]. Moreover, Laino et al. demonstrated that c-Kit expression was found in human adult dental pulp stem cells [20].

Other markers expressed by DPSCs are CD29 and CD44 [25], as well as CD73 and CD105 [26], all markers of mesenchymal stemness. Several DPSCs clones were established from CD271^+^ pulp cells; these CD271^+^ pulp cells also express CD105 and Notch2 [27]. Interestingly, CD271, one of two receptor types for the neurotrophins, a family of protein growth factors that stimulate neuronal cells to survive, was reported to inhibit the differentiation of mesenchymal stem cells, including DPSCs, into osteogenic, adipogenic, chondrogenic, and myogenic lineages [28].

DPSCs are also reported to express OCT4 and Nanog, transcriptional factors involved in pluri/multipotency maintenance [29]. Numerous studies have evaluated the multipotency of DPSCs, which were demonstrated to be able to differentiate in many cell types, such as osteoblasts, smooth muscle cells, adipocytes and neuronal-like cells [30-34]. So far, no cloning based on single surface marker has been capable of isolating cells that match the minimal criteria of MSCs, from different tissue environments. A variety of candidate MSC surface antigens or markers likely related to their stemness have been proposed until now, nevertheless there is a huge difference in their expression in distinct sources of MSCs. As a matter of fact, the identity of MSCs *in vivo* is still unclear, although reports have suggested they may have a fibroblastic or pericytic origin [35,36].

This study was aimed to analyze and compare the characteristics of two subpopulations of hDPSCs. Starting from a first positive immune-selection for STRO-1 and c-Kit (CD117) surface antigens, the sorted STRO-1^+^/c-Kit^+^ hDPSCs underwent a further immune-selection for CD34, in order to separate and compare the STRO-1^+^/c-Kit^+^/CD34^−^ and STRO-1^+^/c-Kit^+^/CD34^+^ sub-fractions, in terms of proliferation capacity, stemness maintenance, multi-lineage differentiation potential, senescence and apoptosis.

As described by Simmons and Torok-Storb [37], CD34 is a typical marker for primitive pluripotent stem cells, both stromal and hematopoietic. Based on the “consensus” extrapolated from the minimal criteria for definition of MSCs, as proposed by The Mesenchymal and Tissue Stem Cell Committee of the International Society for Cellular Therapy [38] CD34 is assumed to be a negative marker for MSCs. On the other hand, CD34 is a universally accepted hematopoietic stem cell (HSC) marker. However, in 1996 Osawa et al. [39] reported the identification of CD34 negative HSCs and that, despite being CD34 negative, these cells remained capable of reconstituting the lymphohematopoietic system. Over the years, extensive research reported the expression of CD34 also by mesenchymal stem cells, obtained from different sources, such as bone marrow mesenchymal stem cells (BM-MSC) [37], adipose derived stem cells (ADSC) [40] and DPSC [41]. According to findings from Laino et al. [42], CD34 expression associated with c-Kit and STRO-1 expression could allow the identification of a niche of hDPSCs derived from neural crest. Though, the function of CD34 is still uncertain. Therefore, it is interesting to isolate the two hDPSCs populations sorted and enriched for STRO-1 and c-Kit expression, associated or not to CD34 expression, and to compare the eventual differences between these two stem cell populations obtained from the same individual.

On the basis of the combined expression of STRO-1, c-Kit and CD34, the STRO-1^+^/c-Kit^+^/CD34^+^ hDPSCs might represent a population of stromal stem cells of neural crest origin. This hypothesis would be in accordance with previous reports whereby head and neck hard tissues of the body have been shown to have, other than a mesodermal origin, a neural crest derivation [20,43].

From these investigations it was found that STRO-1^+^/c-Kit^+^/CD34^−^ hDPSCs and STRO-1^+^/c-Kit^+^/CD34^+^ hDPSCs actually are two different cell populations showing distinct behaviors with regard to cell proliferation rate, stemness maintenance and cell senescence/apoptosis upon late passages. Moreover, differentiation assays performed *in vitro* towards mesoderm (osteogenic, adipogenic, myogenic) and ectoderm (neurogenic) lineages revealed the most evident differences between the two hDPSCs populations; in particular, while no significant differences between the two subpopulations have arisen after differentiation towards the mesoderm lineages (osteogenic, adipogenic, myogenic), the STRO-1^+^/c-Kit^+^/CD34^+^ hDPSCs showed a stronger tendency towards the neurogenic commitment, compared to the STRO-1^+^/c-Kit^+^/CD34^−^ hDPSCs. These data suggest that within dental pulp actually more than a single stem cell population may exist; indeed, stem cells obtained from dental pulp may derive either from mesoderm either from neuro-ectoderm [44,45]. The results obtained in this study might trigger further analyses aimed to better define the flexibility of application of dental pulp derived stem cells for their use in therapeutic applications.

## Methods

### Cell isolation and sorting

Human dental pulp was extracted from the enclosed third molar of teenage subjects undergoing a routine tooth extraction, after written informed consent of their parents (harvested specimen would be discarded anyway). Cells were isolated from dental pulp as described in a previous study [46]. Briefly, dental pulp was harvested from the teeth and immersed in a digestive solution (3 mg/mL type I collagenase plus 4 mg/mL dispase in α-MEM) for 1 h at 37°C. After enzymatic disaggregation, pulp was dissociated and then filtered onto 100 μm Falcon Cell Strainers, in order to obtain a cell suspension. Cell suspension was then plated in 25 cm^2^ flasks and cultured in culture medium [α-MEM with 10% heat inactivated foetal calf serum (FCS), 2 mM L-glutamine, 100 U/mL penicillin, 100 μg/mL streptomycin], at 37°C and 5% CO_2_. At day 3–4 of culture, cells obtained from the entire dental pulp were subsequently trypsinized, resuspended and plated at clonal density (1.6 cell/cm^2^). At day 7, eight cell populations were isolated from colonies originated by single cells. After isolation of formed colonies, hDPSCs were expanded upon reaching 70% confluency and about 5 × 10^6^ cells were used for following magnetic cell sorting (passage 2).

The STRO-1^+^/c-Kit^+^ hDPSCs were obtained by magnetic cell sorting (MACS; Miltenyi Biotec) using a mouse anti-STRO-1 and a rabbit anti-c-Kit antibodies (Abs; Santa Cruz). This hDPSCs population underwent a further immune-selection by MACS technology using a mouse anti-CD34 ab (Millipore), in order to separate the STRO-1^+^/c-Kit^+^/CD34^−^ and STRO-1^+^/c-Kit^+^/CD34^+^ fractions.

### FACS analysis

In order to assay the percentage of cells expressing STRO-1, c-Kit and CD34 surface antigens, FACS analysis was performed on the whole unsorted hDPSCs after *in vitro* expansion (~5 × 10^6^ cells), at passage 1. Likewise, the percentage of cells triple labelled for STRO-1, c-Kit and CD34 or double labelled for STRO-1, c-Kit and negative for CD34 expression was evaluated.

Indirect staining was performed using mouse IgM anti-STRO-1, rabbit IgG anti-c-Kit (Santa Cruz) and mouse IgG anti-CD34 (Millipore), followed by goat anti-mouse-IgM-Alexa488, donkey anti-rabbit-IgG-Alexa647, and goat anti-mouse-IgG-Alexa405 (Invitrogen). Non-specific fluorescence was assessed by using normal mouse IgG or IgM followed by the secondary antibody as described above. Cells were acquired using Attune acoustic flow cytometer (Life Technologies, Thermo Fischer Scientific) equipped with four lasers (blue 488 nm, yellow 561 nm, red 638 nm and violet 405 nm). Data were analyzed using FlowJo 9.8 (Treestar, Miltenyi).

### Cell proliferation

The proliferation rate was analyzed on both CD34^−^ and CD34^+^ hDPSCs populations, seeded at the density of 4 × 10^3^ cells/cm^2^ and cultured for 1 week until reaching the confluence.

Each day cell counting was performed on both CD34^−^ and CD34^+^ hDPSCs. The mean of cell number was calculated on three experimental samples for each experimental group and cell density was expressed as mean of cells/cm^2^ ± standard deviation (SD). The population doubling time (PDT) was calculated in the phase of exponential growth by the following formula:

$$ PDT=\frac{lo{g}_{10}(2)\times \varDelta T}{lo{g}_{10}\left({N}_{7d}\right)\mathit{\hbox{-}}lo{g}_{10}\left({N}_{1d}\right)} $$

*N*_7d_ is the cell number at day 7 and *N*_1d_ is the cell number at day 1. Then, to determine the population doubling (PD) rate, both CD34^−^ and CD34^+^ hDPSCs populations were initially seeded in culture medium at the density of 4 × 10^3^ cells/cm^2^. Cells were passaged and counted once they reached a sub-confluence of 80%. At each passage cell were re-plated at the initial density and cultures were performed until passage 6. Three samples for each experimental group were used. The following formula was applied:

$$ PD=\frac{lo{g}_{10}(N)\mathit{\hbox{-}}lo{g}_{10}\left({N}_s\right)}{lo{g}_{10}(2)} $$

*N* is the harvested cell number and *N*_s_ is the initial plated cell number. Cumulative population doublings (CPD) index for each passage was obtained by adding the PD of each passage to the PD of the previous passages [47].

### Cell senescence and apoptosis

In order to investigate cell senescence occurrence in CD34^−^ and CD34^+^ hDPSCs, cells at passage 6 were seeded in 12-well plates and cultured upon confluence. Samples were then processed by a senescence β-galactosidase staining kit (Cell Signaling), according to manufacturer’s instructions. Three samples for each hDPSCs population were analyzed and the percentage of senescent cells was calculated. The presence of apoptotic cells in CD34^−^ and CD34^+^ hDPSCs cultures was analyzed by detection of the active form of caspase 3. Whole cell lysates of CD34^−^ and CD34^+^ hDPSCs at passage 1 and 6 were processed for Western blot analysis and active caspase 3 was detected by an anti-caspase 3 specific ab (Cell Signaling). Densitometry of active caspase 3 bands was performed by NIS software (Nikon). An equal area was selected inside each band and the mean of gray levels (in a 0–256 scale) was calculated. Data, represented as integrated density (the sum of pixel values minus a background value for each pixel within a bounded area), were then normalized to values of background and of control actin band.

### Surface antigens expression: confocal immunofluorescence analysis

The expression of stemness surface antigens by hDPSCs from both CD34^−^ and CD34^+^ populations was assessed at passage 1 and 6 through immunofluorescence labelling. Monolayer cells were fixed in 4% paraformaldehyde in phosphate buffer saline (PBS) at pH 7.4 for 20 minutes, then after washing in PBS, cells were permeabilized with 0,1% Triton X-100 in PBS for 5 minutes; samples were then blocked with 3% BSA in PBS for 30 minutes at room temperature and then incubated with the primary antibodies diluted 1: 50 [mouse IgM anti-STRO-1 (Santa Cruz), rabbit anti-c-Kit (Santa Cruz), mouse anti-CD34 (Millipore)] in PBS containing 3% BSA, for 1 hour at room temperature. After washing in PBS containing 3% BSA, the samples were incubated for 1 hour at room temperature with the secondary antibodies diluted 1:200 in PBS containing 3% BSA (goat anti-mouse Alexa647; goat anti-rabbit Alexa488; donkey anti-mouse Alexa546). After washing in PBS samples were stained with 1 μg/ml DAPI in PBS for 1 minute, and then mounted with anti-fading medium (FluoroMount, Sigma Aldrich). Negative controls consisted of samples not incubated with the primary ab. The multi-labelling immunofluorescence experiments were carried out avoiding cross-reactions between primary and secondary abs. Confocal imaging was performed by a Nikon A1 confocal laser scanning microscope as previously described [48,49]. The confocal serial sections were processed with ImageJ software to obtain three-dimensional projections and image rendering was performed by Adobe Photoshop Software.

### Multilineage differentiation

The CD34^−^ and CD34^+^ hDPSCs populations were also evaluated for their multilineage differentiation potential, namely they were induced towards osteogenic, myogenic, adipogenic and neurogenic lineages. Three samples per experimental group were used for each differentiation experiment.

In order to obtain osteogenic differentiation CD34^−^ and CD34^+^ hDPSCs were seeded at approximately 3 × 10^3^ cells/cm^2^ on culture dishes in osteogenic medium (culture medium supplemented with 5% FCS, 100 μM 2P-ascorbic acid, 100 nM dexamethasone, 10 mM β-glycerophosphate). Double immunofluorescence stainings, by using rabbit anti-Runx2 (Abcam) and mouse anti-osteopontin (OPN; Santa Cruz) abs, rabbit anti-osterix (Osx; GeneTex) and mouse anti-osteocalcin (OCN; Abcam) abs, were performed to evaluate the expression of bone specific proteins, four weeks after osteogenic induction on both hDPSCs subpopulations. Moreover deposition of mineralized extracellular matrix was assessed through Alizarin Red staining.

Myogenic differentiation experiments were carried out as formerly described by Pisciotta et al. [47]: the two hDPSCs subpopulations were directly co-cultured with C2C12 mouse myoblasts with a 10:1 seeding ratio, in DMEM High Glucose, supplemented with 10% FCS, 2 mM L-glutamine, 100 U/ml penicillin, 100 mg/ml streptomycin, until confluence was reached. Upon confluence, growth medium was replaced by DMEM High Glucose supplemented with 1% FCS and 10 nM insulin. Cells were maintained in co-culture for 2 weeks. Direct co-culture with C2C12 myoblasts was performed to evaluate the myogenic potential of the two hDPSCs subpopulations, since this practice represents an *in vitro* model of myogenesis to test the ability of non-myogenic cell types to fuse and form new myotubes [42,50].

Double immunofluorescence stainings, by using a mouse anti-human mitochondrial protein ab (anti-hMit; Millipore) or a mouse anti-human Nuclei ab (hNu; Millipore) and a rabbit anti-myosin (Sigma Aldrich) ab, were performed in order to verify the formation of myotubes with the direct contribution of the two hDPSCs subpopulations.

Adipogenic and neurogenic differentiation were performed as previously described [47]. Adipogenic differentiation: CD34^−^ and CD34^+^ hDPSCs were seeded on 24-well plates at a cell density of 2 × 10^4^ cells/cm^2^. Subconfluent cultures were incubated in the adipogenic medium (culture medium medium containing 0.5 mM isobutyl-methylxanthine, 1 μM dexamethasone, 10 μM insulin, 200 μM indomethacin, 50 mg/mL gentamicin) for 3 weeks. Medium was changed every 3 days. Afterwards, cells were evaluated for the formation of lipid droplets by means of oil red O staining and AdipoRed assay (according to manufacturer’s instructions; Lonza).

Neurogenic differentiation: both the hDPSCs populations were seeded on 6-well plates at 2 × 10^4^ cells/cm^2^ cell density. After confluency was reached, cells were pre-induced with culture medium supplemented with 1 mM β-mercaptoethanol. After 24 hours, the cells were washed in PBS and differentiated in serum free α-MEM containing 10 mM β-mercaptoethanol, 2% dimethyl sulfoxide and 200 μM butylated hydroxyanisole until neuronal-like morphology was appreciable. Both hDPSCs populations were assayed for the expression of either neuronal specific markers, such as β-III-Tubulin, MAP2, Neu-N synapsin, and of glial specific markers, such as GFAP.

### Expression of nestin and CD271

Nestin, a member of the intermediate filament proteins family, is expressed in dividing cells during the early stages of development in the central nervous system, while the cell surface antigen CD271 is one of the two receptor types for neurotrophins, a family of protein growth factors that promote neuronal cells survival and differentiation. The expression of these markers was evaluated through immunofluorescence labelling with mouse anti-nestin (Millipore) and mouse anti-CD271 (BioLegend) abs in both undifferentiated CD34^−^ and CD34^+^ hDPSCs subpopulations.

### Histochemistry and western blot analysis

Three samples of *in vitro* differentiated CD34^−^ and CD34^+^ hDPSCs were fixed in 4% paraformaldehyde in PBS at pH 7.4 for 20 minutes and were processed for subsequent steps. For Alizarin red staining fixed cells were incubated for 5 minutes at room temperature in a solution containing 0.1% alizarin red and 1% ammonium hydroxide. Images were collected by a CCD colour camera equipped with a 90 mm macro photograph objective.

Densitometry was performed on culture plates from three independent experiments by NIS software (Nikon). An equal area (ROI) was selected around the plate surface and the mean of gray levels (in a 0–256 scale) was calculated. Data were then normalized to values of background and expressed as mean ± SD.

Whole cell lysates obtained from both hDPSCs populations, CD34^−^ and CD34^+^, differentiated in osteogenic medium, were processed for Western blot analysis as previously described [48,49].

Thirty μg of protein extract from each sample were separated by SDS-PAGE and then transferred to PVDF membranes. The following abs were used: mouse anti-osteocalcin (OCN, Abcam); mouse anti-human Collagen I (Millipore); rabbit anti-caspase 3 (Sigma Aldrich) and rabbit anti-β-III-tubulin (Cell Signaling) diluted 1:1000; HRP-conjugated anti-mouse and anti-rabbit secondary ab (Pierce Antibodies, Thermo Scientific) diluted 1:3000. The membranes were visualized by using Enhanced Chemio Luminescence (ECL; Amersham). Anti-actin ab was used as control of protein loading. Densitometry of OCN and Collagen I was performed by NIS software (Nikon). An equal area was selected inside each band and the mean of gray levels (in a 0–256 scale) was calculated. Data were then normalized to values of background and of control actin band. List of antibodies used and related dilutions were reported in Table 1.

**Table 1:** **List of antibodies and related dilution used**

**Name**	**Host**	**Provider**	**Dilution**	**Application**
anti-STRO-1	mouse	Santa Cruz	1: 50	FACS, IF
anti-c-Kit	rabbit	Santa Cruz	1: 50	FACS, IF
anti-CD34	mouse	Millipore	1: 50	FACS, IF
anti-Runx2	rabbit	Abcam	1:100	IF
anti-osteopontin	mouse	Santa Cruz	1:100	IF
anti-osterix	rabbit	GeneTex	1:100	IF
anti-osteocalcin	mouse	Abcam	1:100; 1:1000	IF; WB
anti-human collagen I	mouse	Millipore	1:1000	WB
anti-human nuclei	mouse	Millipore	1:100	IF
anti-human mitochondrial protein	mouse	Millipore	1:100	IF
anti-myosin	rabbit	Sigma Aldrich	1:100	IF
anti-β-III-Tubulin	rabbit	Cell Signaling	1:100; 1:1000	IF; WB
anti-MAP2	mouse	Sigma Aldrich	1:100; 1:1000	IF; WB
anti-NeuN	mouse	Millipore	1:100	IF
anti-Synapsin	rabbit	Cell Signaling	1:100	IF
anti-GFAP	mouse	Cell Signaling	1:100; 1:1000	IF; WB
anti-nestin	mouse	Millipore	1:100	IF
anti-CD271	mouse	BioLegend	1:100	IF
anti-caspase 3	rabbit	Sigma Aldrich	1:1000	WB
anti-actin	goat	Santa Cruz	1: 3000	WB

### Statistical analysis

All experiments were performed in triplicate. Data were expressed as mean ± standard deviation (SD). Differences between the two experimental groups were analyzed by paired, Student’s *t* test. Differences among three or more experimental samples were analyzed by ANOVA followed by Newman-Keuls post-hoc test (GraphPad Prism Software version 5 Inc., San Diego, CA, USA). In any case, significance was set at *p*<0.05.

## Results

### FACS analysis

**Figure 1:**
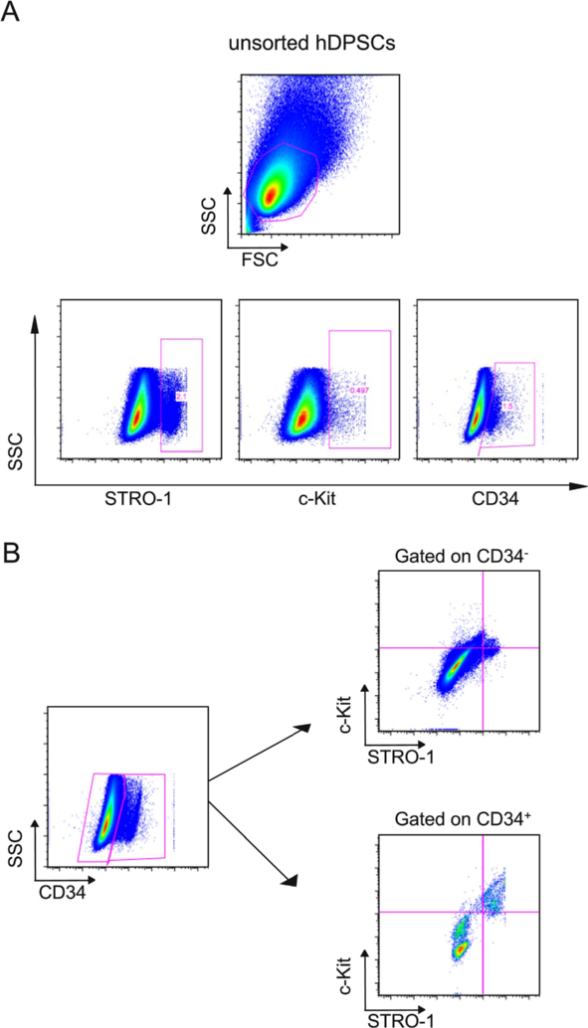
**Cytofluorimetric analysis of STRO-1, c-Kit and CD34 expression in whole unsorted hDPSCs. A**: Unsorted hDPSCs were plotted SSC vs. STRO-1, c-Kit and CD34 at day 7after isolation and expansion *in vitro*. **B**: unsorted hDPSCs were gated on CD34, then the plot STRO-1 vs c-Kit was done in CD34+ and CD34- cells.

The expression of STRO-1, c-Kit and CD34 surface antigens was assayed on unsorted hDPSCs after *in vitro* expansion, at passage 1. Flow cytometry analysis revealed that the unsorted hDPSCs showed the expression of the three evaluated markers, specifically ~ 2.1% STRO-1, ~ 0.5% c-Kit and ~ 1.5% CD34, respectively (Figure 1A).

Furthermore, flow cytometry analysis was performed to evaluate the percentage of cells simultaneously positive for the three markers STRO-1/c-Kit/CD34 and cells positive for STRO-1/c-Kit and negative for CD34. Data showed that ~ 0.5% hDPSCs negative for CD34 co-expressed STRO-1 and c-Kit, whereas ~ 20% of hDPSCs positive for CD34 co-expressed STRO-1 and c-Kit (Figure 1B).

### Cell proliferation

**Figure 2:**
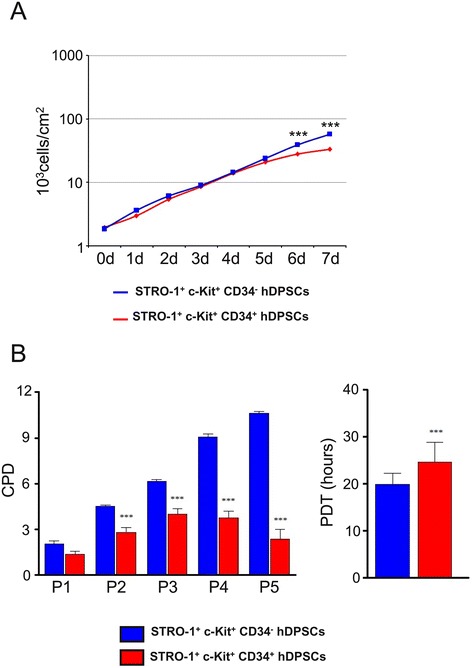
**Evaluation of cell proliferation in STRO-1** ^**+**^ **/c-Kit** ^**+**^ **/CD34** ^**−**^ **hDPSCs and STRO-1** ^**+**^ **/c-Kit** ^**+**^ **/CD34** ^**+**^ **hDPSCs. A**: Proliferation rate of STRO-1^+^/c-Kit^+^/CD34^−^ hDPSCs and STRO-1^+^/c-Kit^+^/CD34^+^ hDPSCs cultured for 1 week. Values, expressed as mean ± SD, are reported in a Log scale, n = 3; *indicates values of unpaired *t*-test STRO-1^+^/c-Kit^+^/CD34^+^ hDPSCs vs STRO-1^+^/c-Kit^+^/CD34^−^ hDPSCs (***p<0,001). **B**: Left side: cumulative population doubling (CPD) of STRO-1^+^/c-Kit^+^/CD34^−^ hDPSCs and STRO-1^+^/c-Kit^+^/CD34^+^ hDPSCs cultured for a total of 5 passages (n = 3; ***p<0,001). Right side: population doubling time (PDT) of STRO-1^+^/c-Kit^+^/CD34^−^ hDPSCs and STRO-1^+^/c-Kit^+^/CD34^+^ hDPSCs was calculated in log phase. Values are reported as mean ± SD; *indicates values of unpaired *t*-test STRO-1^+^/c-Kit^+^/CD34^+^ hDPSCs vs STRO-1^+^/c-Kit^+^/CD34^−^ hDPSCs (***p<0,001).

In order to evaluate the growth kinetics of both CD34^−^ and CD34^+^ hDPSCs, proliferation rate was analyzed on both the populations seeded in 60 mm Petri dishes at the density of 4 × 10^3^ cells/cm^2^ and cultured for 1 week until reaching the confluence. Cell counting was performed each day on 6 randomly selected fields of 1 mm^2^. Both the cell populations, CD34^−^ and CD34^+^ hDPSCs demonstrated a parallel rising trend up to day 5, then, differences in growth kinetics of these two populations were observed; in particular, the CD34^−^ hDPSCs showed a consistent increase in proliferation upon day 8 without arresting, while the CD34^+^ hDPSCs population dramatically slowed down from day 6 through day 8 (Figure 2A). As a matter of fact, the proliferation index of CD34^+^ hDPSCs was significantly lower at days 7 and 8 when compared to the CD34^−^ hDPSCs population (Figure 2A). Statistically significant differences between the two hDPSCs populations were highlighted by the analysis of cumulative population doubling (CPD) which, as early as at passage 2 (P2), resulted significantly lower in CD34^+^ population compared to CD34^−^ population, a significant difference that was observed through the passages up to P5 (Figure 2B, left side). Data from CPD analysis demonstrated also that CD34- hDPSCs had a steadily rising growth kinetics through all the passages, whereas the CD34^+^ hDPSCs reached a growth peak at P3, then slowed down at P4 up to P5 (Figure 2B, left side). Furthermore, the population doubling time (PDT) of CD34^−^ hDPSCs was lower than CD34^+^ hDPSCs (STRO-1^+^/c-Kit^+^/CD34^−^ hDPSCs: 19.91± 2.30 hours; STRO-1^+^/c-Kit^+^/CD34^+^ hDPSCs: 24.55 $\pm$ 4.20 hours; ***p<0.001) (Figure 2B, right side).

### Cell senescence, apoptosis and surface antigens expression

**Figure 3:**
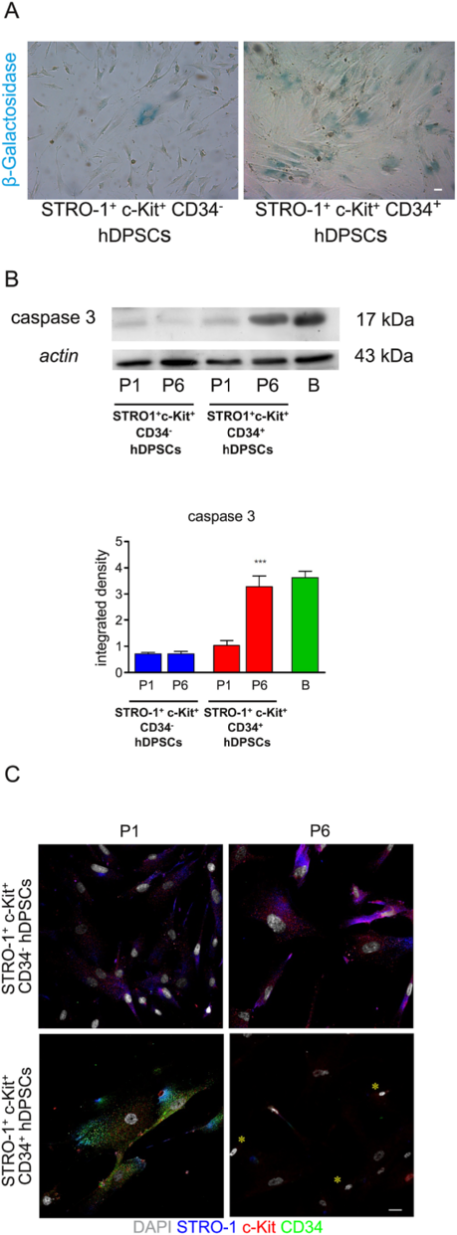
**Cell senescence, apoptosis and surface antigens expression in STRO-1** ^**+**^ **/CD34** ^**−**^ **hDPSCs and STRO-1** ^**+**^ **/c-Kit** ^**+**^ **/CD34** ^**+**^ **hDPSCs. A**: β-Galactosidase activity staining in confluent cultures of STRO-1^+^/c-Kit^+^/CD34- hDPSCs and STRO-1^+^/c-Kit^+^/CD34^+^ hDPSCs grown for 5 passages. Blue labeling indicates cells positive to β-galactosidase activity staining. Bar: 50 μm. **B**: Western blot analysis of active caspase 3 expression by STRO-1^+^/c-Kit^+^/CD34^−^ hDPSCs and STRO-1^+^/c-Kit^+^/CD34^+^ hDPSCs at passages 1 and 6. Blasts treated with etoposide were loaded as positive control of the presence of active caspase 3. Actin bands were presented as control of the protein loading. Densitometry of caspase 3 bands was shown at the bottom of western blot images. [***p<0,001 DPSC STRO-1^+^/c-Kit^+^/CD34^+^ (P6) vs DPSC STRO-1^+^/c-Kit^+^/CD34^+^ (P1), unpaired *t*-test; n=3]. **C**: Immunofluorescence labeling shows signals from c-Kit (red), STRO-1 (blue) and CD34 (green) in both the subpopulations of hDPSCs, analyzed at passages 1 and 6. DAPI staining is shown in grey. Yellow asterisks indicate the presence of pyknotic nuclei. Bar: 10 μm.

Cell senescence, evaluated by detection of β-galactosidase activity in confluent culture of CD34^−^ and CD34^+^ hDPSCs grown up to 5 passages, revealed very low levels of β-galactosidase in CD34^−^ population, whereas a significant percentage of senescent cells was revealed in CD34^+^ population by microscopic observation (Figure 3A; <2% STRO-1^+^/c-Kit^+^/CD34^−^ hDPSCs vs ≈ 90% STRO-1^+^/c-Kit^+^/CD34^+^ hDPSCs positive to β-galactosidase assay).

Moreover, western blot analysis of active caspase 3 was carried out on whole cell lysates from CD34^−^ and CD34^+^ hDPSCs at passage 1 and 6 to evaluate whether apoptosis occurred at late passage (Figure 3B, top). A significant amount (p<0.001) of the active form of caspase 3 (17 kDa) was expressed by CD34^+^ hDPSCs obtained at passage 6, conversely no significant expression of caspase 3 could be detected in CD34^−^ hDPSCs, as shown by densitometric analysis (Figure 3B, bottom).

Human DPSCs from both the CD34^−^ and CD34^+^ populations were obtained at passage 1 and 6 and evaluated through immunofluorescence labeling for the expression of the markers they were immune-selected for. While the expression of the above mentioned antigens was preserved by the CD34^−^ subpopulation either at passage 1 and passage 6, a reduction in the expression of all the three antigens was observed in the CD34^+^ subpopulation, as early as at passage 6 (Figure 3C). Moreover, the presence of pyknotic nuclei, which denotes a degenerative process of cell nucleus, occurring in apoptosis and necrosis, with the nucleus showing a reduction in volume and becoming more intensely stained, was observed in CD34^+^ subpopulation at late passages (Figure 3C, yellow asterisks).

### Multilineage differentiation

Human DPSCs from both CD34^−^ and CD34^+^ subpopulations were differentiated towards osteogenic, myogenic, adipogenic and neurogenic lineages, as described in materials and methods section.

**Figure 4:**
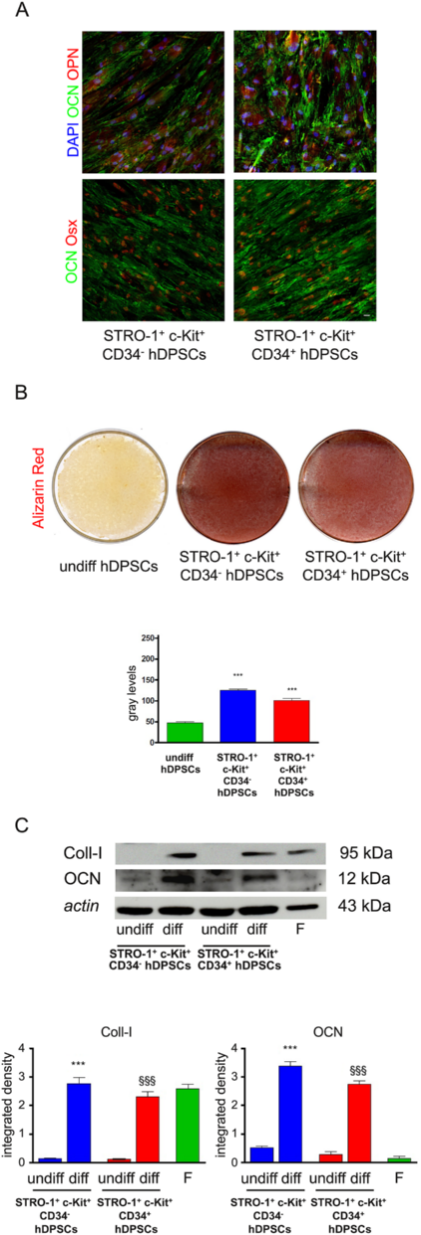
**Evaluation of osteogenic differentiation of STRO-1** ^**+**^ **/c-Kit** ^**+**^ **/CD34** ^**−**^ **hDPSCs and STRO-1** ^**+**^ **/c-Kit** ^**+**^ **/CD34** ^**+**^ **hDPSCs. A**: Confocal analysis of osteogenic differentiation of STRO-1^+^/c-Kit^+^/CD34^−^ hDPSCs and STRO-1^+^/c-Kit^+^/CD34^+^ hDPSCs. Double immunofluorescence confocal images showing signals from anti-OPN (red), anti-OCN (green) and DAPI (blue), and from anti-OCN (green) and anti-Osx (red), respectively. Bar: 10 μm. **B**: Alizarin Red staining on STRO-1^+^/c-Kit^+^/CD34^−^ hDPSCs and STRO-1^+^/c-Kit^+^/CD34^+^ hDPSCs. Densitometric analysis show the deposition of extracellular mineralized matrix by both the subpopulations after the osteogenic induction. Values are mean ± SD of gray levels (0–255 scale). STRO-1^+^/c-Kit^+^/CD34^−^ hDPSCs n = 3; STRO-1^+^/c-Kit^+^/CD34^+^ hDPSCs n = 3; *** *p*<0,001 STRO-1^+^/c-Kit^+^/CD34^−^ hDPSCs and STRO-1^+^/c-Kit^+^/CD34^+^ hDPSCs vs undifferentiated hDPSCs, ANOVA test followed by Newman-Keuls post-hoc test. **C**: Western blot (WB) analysis of Coll-I and OCN expression in whole cell lysates of differentiated STRO-1^+^/c-Kit^+^/CD34^−^ hDPSCs and STRO-1^+^/c-Kit^+^/CD34^+^ hDPSCs. Whole cell lysates were collected from three plates of hDPSCs for each experimental group. Fibroblasts were used as positive controls for Coll-I expression and as negative controls for osteocalcin expression, respectively. Actin bands demonstrate that an equal amount of protein was loaded in each line. Densitometric analysis of the bands corresponding to Coll-I and OCN is shown below western blot images. Values are mean ± SD of gray levels (0–255 scale); ***p<0,001 diff STRO-1^+^/c-Kit^+^/CD34^−^ hDPSCs vs undiff STRO-1^+^/c-Kit^+^/CD34^−^, ^§§§^ *p*<0,001 diff STRO-1^+^/c-Kit^+^/CD34^+^ hDPSCs vs undiff STRO-1^+^/c-Kit^+^/CD34^+^ hDPSCs, ANOVA test followed by Newman-Keuls post-hoc test.

After 4 weeks of osteogenic induction in both CD34^−^ and CD34^+^ hDPSCs cultures confocal analysis revealed the expression of osteocalcin (OCN), together with other specific markers of osteogenic commitment, such as osterix (Osx) and osteopontin (OPN) (Figure 4A). Double immunofluorescence labeling was aimed to simultaneously analyze the localization of OCN/Osx and OPN/OCN in both hDPSCs populations (Figure 4A). Signal from OCN, which appeared typically localized in the cytoplasm, was also detected in extracellular matrix, where it was present as spots corresponding to mineralization areas. Conversely, Osx showed a peculiar nucleoplasmic localization. No significant differences were observed between the two hDPSCs subpopulations. Mineralization of the extracellular matrix, detected through Alizarin Red staining, was clearly evident in both CD34^−^ and CD34^+^ subpopulations (Figure 4B); no significant differences were observed between the two experimental groups. A further confirmation that both CD34^−^ and CD34^+^ hDPSCs achieved the osteogenic commitment was provided by the expression of OCN and type I collagen (Coll-I) through western blot analysis on whole cell lysates of differentiated hDPSCs. No significant differences between the two populations were detected by densitometry.

**Figure 5:**
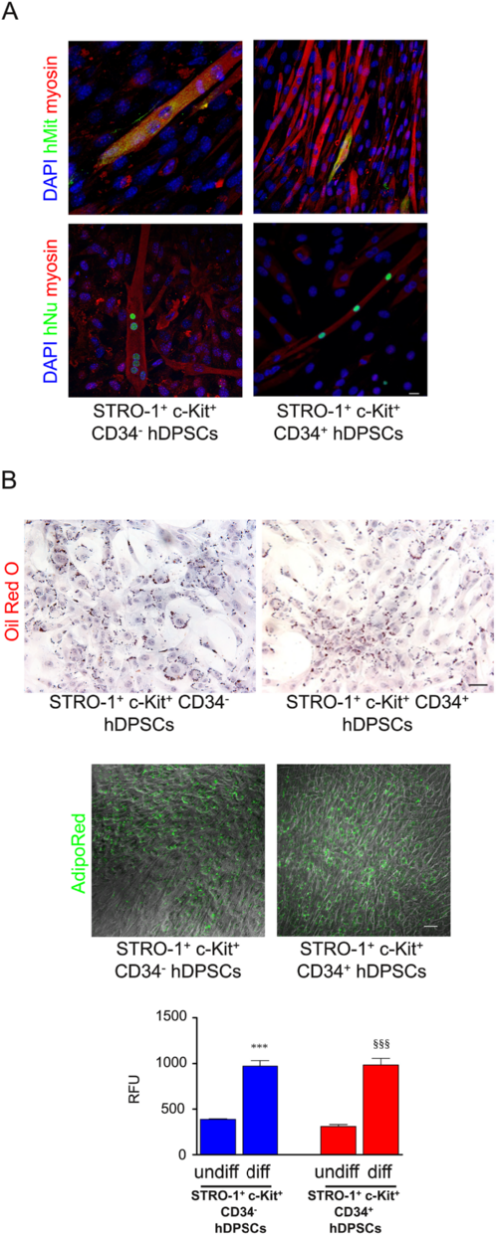
**Evaluation of myogenic differentiation and adipogenic differentiation in STRO-1** ^**+**^ **/c-Kit** ^**+**^ **/CD34** ^**−**^ **hDPSCs and STRO-1** ^**+**^ **/c-Kit** ^**+**^ **/CD34** ^**+**^ **hDPSCs. A**: Confocal analysis of myogenic differentiation of STRO-1+/c-Kit+/CD34^−^ hDPSCs and STRO-1^+^/c-Kit^+^/CD34^+^ hDPSCs directly co-cultured with C2C12 mouse myoblasts. Double immunofluorescence labeling shows signals from anti-hMit (green) and anti-myosin (red) and from anti-hNu (green) and anti-myosin (red), respectively. DAPI staining is shown in blue. Bar: 10 μm. **B**: Evaluation of adipogenic commitment in STRO-1^+^/c-Kit^+^/CD34^−^ hDPSCs and STRO-1^+^/c-Kit^+^/CD34^+^ hDPSCs. On the top, histological staining with Oil Red O showing adipogenic differentiation of STRO-1^+^/c-Kit^+^/CD34^−^ hDPSCs and STRO-1^+^/c-Kit^+^/CD34^+^ hDPSCs. Cells were counterstained with haematoxylin. Bar: 10 μm. Below, AdipoRed assay performed on both hDPSCs subpopulations, evaluated for lipid droplets formation by confocal microscopy analysis. Differentiated STRO-1^+^/c-Kit^+^/CD34^−^ hDPSCs and STRO-1^+^/c-Kit^+^/CD34^+^ hDPSCs show a green fluorescent staining. Bar: 10 μm. At the bottom, quantification of triglycerides (RFU, relative fluorescent unit) carried out by fluorimeter analysis. ***p<0,001 diff STRO-1^+^/c-Kit^+^/CD34^−^ hDPSCs vs undiff STRO-1^+^/c-Kit^+^/CD34^−^ hDPSCs; ^§§§^ *p*<0,001 diff STRO-1^+^/c-Kit^+^/CD34^+^ hDPSCs vs undiff STRO-1^+^/c-Kit^+^/CD34^+^ hDPSCs, ANOVA test followed by Newman-Keuls post-hoc test.

The ability of CD34^−^ and CD34^+^ hDPSCs to differentiate towards myogenic lineage was assessed by direct co-culture with C2C12 mouse myoblasts. After 2 weeks of co-culture myotubes formation was observed in co-cultures with C2C12 myoblasts and hDPSCs from both CD34^−^ and CD34^+^ subpopulations. Myotubes appeared multinucleated indicating that cell fusion occurred. Labeling by anti-human mitochondria antibody (anti-hMit) demonstrated that both hDPSCs subpopulations were involved in myotubes generation. The expression of myosin by newly formed myotubes indicated that a terminal myogenic commitment was reached (Figure 5A). Double immunofluorescence staining with anti-human nuclei (hNu) and anti-myosin antibodies demonstrated that in both culture conditions mature myotubes were formed with the contribution of hDPSCs. Myotubes not labeled by anti-hMit/hNu antibody and therefore formed only by C2C12 cells were also present (Figure 5A).

For adipogenic differentiation CD34^−^ and CD34^+^ hDPSCs started to form lipid droplets after 10 days of induction. After 3 weeks of differentiation, lipid droplets formation was evaluated either by oil red O staining and AdipoRed assay (Figure 5B). Oil red O staining revealed by microscopic observation the formation of lipid droplets by both CD34^−^ and CD34^+^ hDPSC populations (Figure 5B, top). AdipoRed assay was performed in order to confirm the data from microscopic observation; as AdipoRed binding to triglycerides contained within intracellular lipid rich vacuoles leads to fluorescence emission, both hDPSCs subpopulations were evaluated for lipid droplets formation by confocal microscopy analysis, showing a fluorescent staining (Figure 5B, middle). Also, quantification of triglycerides - expressed as relative fluorescent unit (RFU) - carried out by fluorimeter analysis confirmed the adipogenic commitment of both CD34^−^ and CD34^+^ hDPSCs populations (STRO-1^+^/c-Kit^+^/CD34^−^ hDPSCs: 971.9 ± 61.0 RFU; STRO-1^+^/c-Kit^+^/CD34^+^ hDPSCs: 984.0 ± 72.7 RFU). No differences were observed between the two experimental groups (Figure 5B, bottom).

**Figure 6:**
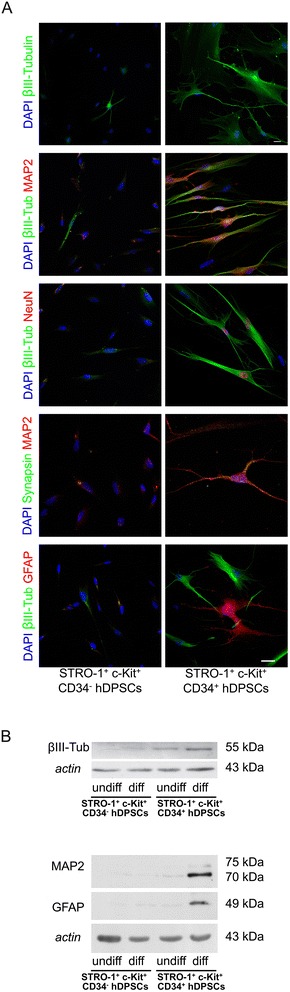
**Neurogenic differentiation of STRO-1** ^**+**^ **/c-Kit** ^**+**^ **/CD34** ^**−**^ **hDPSCs and STRO-1** ^**+**^ **/c-Kit** ^**+**^ **/CD34** ^**+**^ **hDPSCs. A**: Confocal analysis of neurogenic differentiation of STRO-1^+^/c-Kit^+^/CD34^−^ hDPSCs and STRO-1^+^/c-Kit^+^/CD34^+^ hDPSCs after 1 week of induction. Immunofluorescent staining show differentiated STRO-1^+^/c-Kit^+^/CD34- hDPSCs and STRO-1^+^/c-Kit^+^/CD34^+^ hDPSCs expressing β-III-Tubulin, MAP2, NeuN, Synapsin and GFAP. Bar: 10 μm. **B**: Western blot (WB) analysis of β-III-Tubulin, MAP2 and GFAP expression in whole cell lysates of differentiated STRO-1^+^/c-Kit^+^/CD34^−^ hDPSCs and STRO-1^+^/c-Kit^+^/CD34^+^ hDPSCs. Actin bands were used as control of protein loading.

Neurogenic differentiation started with a first cell detachment from culture plates, event that may be likely induced by β-mercaptoethanol. Cells still adhering progressively assumed a neuronal-like morphology with multiple cellular processes and a defined cell body. After 1 week of induction a strong response was observed in CD34^+^ hDPSCs which showed a greater expression of β-III-Tubulin, compared to CD34^−^ hDPSCs, which demonstrated to be induced only to a lesser extent towards the neurogenic commitment (Figure 6A). Moreover, the expression of further neuronal specific markers was investigated, showing that CD34^+^ hDPSCs were labeled by anti-MAP2, anti-Neu-N and anti-synapsin antibodies, confirming the occurrence of neuronal differentiation. Figure 6A shows that CD34^+^ hDPSCs positive for neuronal marker (β-III-Tubulin) are distributed close to glial cells positively stained by GFAP marker. These results were confirmed by western blot analysis highlighting a difference between the two experimental groups (Figure 6B).

### Expression of nestin and CD271

**Figure 7:**
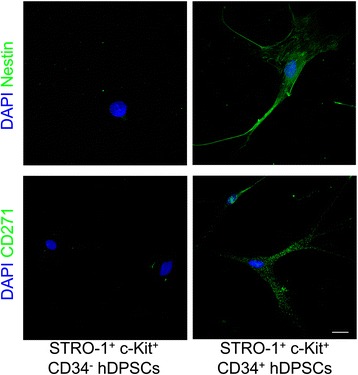
**Expression of nestin and CD271 by undifferentiated hDPSCs subpopulations.** Confocal analysis of the expression of neural markers, nestin and CD271, by undifferentiated STRO-1^+^/c-Kit^+^/CD34^−^ hDPSCs and STRO-1^+^/c-Kit^+^/CD34^+^ hDPSCs. Immunofluorescence labeling shows signals from nestin (green) and CD271 (green), respectively. DAPI staining is shown in blue. Bar: 10 μm.

Immunofluorescence analysis was carried out on undifferentiated CD34^−^ and CD34^+^ hDPSCs in order to evaluate the expression of nestin and CD271, which are involved in the early development of central nervous system and in promotion of survival and differentiation of neuronal cells besides identifying the neural crest derived cells. A clear difference between the two subpopulations was observed, as the CD34^−^ hDPSCs did not express any of these markers, while CD34^+^ hDPSC subpopulation showed the expression of nestin and CD271 (Figure 7).

## Discussion

Mesenchymal cells derived from neural crest are responsible for the construction of craniofacial skeleton during embryo and tooth-periodontium formation over fetal and adult life [51]. These cells are involved in the development of several, although distinct, hard tissues, including crown and root dentin, cementum, and alveolar bone. So far, according to their common origin, it has not been feasible to identify a specific marker for differentiated cells of each of these structures.

Furthermore, neural crest-derived stem cells are also present within the adult human body, enclosed within the dental pulp, which hence represents a source of stromal stem cells [9], contained in an ideal *niche*, i.e. the pulp chamber. Many are the sources from which dental stem cells can be retrieved, such as human permanent teeth (DPSCs), exfoliated deciduous teeth (SHEDs), apical papilla of immature permanent teeth (SCAPs), periodontal ligament (PDLSCs) and dental follicle (DFPCs) [9-16].

Several different mesenchymal stem cell markers were used to select distinct subsets of DPSCs, exhibiting different biological behaviors [18]. Among several surface antigens, three specific stem cell markers were analyzed; STRO-1, considered an early marker of mesenchymal stem cells [52-54], identifies a cell surface antigen expressed by the osteogenic fraction of stromal precursors in human bone marrow as well as in erythroid precursors [52,53,55,56]. Other data have suggested that STRO-1 recognizes also a stromal cell precursor of pericyte cells in dental pulp [57]. Another marker for stem cells, c-Kit, which has been already used for stem cells [58-60], was highly and early expressed in dental pulp stem cells. Interestingly, c-Kit is clearly expressed in neural crest-derived cells, like melanocyte precursors [21] and cells deriving from neural crest progenitors, like dental pulp. Furthermore, CD34, another marker associated to primitive pluripotent stem cells both stromal and hematopoietic [37], has been analyzed.

Notably, increasing evidence has been demonstrating CD34 expression by several cell types, including multipotent mesenchymal stromal cells [61], although a misconception persists that CD34 distinctively represents cells of hematopoietic origin. Given these considerations, a huge interest has been aroused towards the investigation of CD34 expression and role in a specific subpopulation of hDPSCs.

Our data demonstrated that a low percentage of unsorted hDPSCs was positive for the expression of all the three markers. Based on these preliminary considerations, the aim of this study was to analyze and compare the characteristics of two subpopulations of hDPSCs. Starting from a first positive immune-selection for STRO-1 and c-Kit surface antigens, the sorted STRO-1^+^/c-Kit^+^ hDPSCs underwent a further immune-selection for CD34, in order to separate and compare the STRO-1^+^/c-Kit^+^/CD34^−^ and STRO-1^+^/c-Kit^+^/CD34^+^ subpopulations, in terms of proliferation capacity, stemness maintenance, senescence, apoptosis, and multi-lineage differentiation potential. From these investigations it was found that STRO-1^+^/c-Kit^+^/CD34^−^ hDPSCs and STRO-1^+^/c-Kit^+^/CD34^+^ hDPSCs actually are two different cell populations showing distinct behaviors.

Data from the analysis of cell proliferation, with particular regard to CPD and PDT values, demonstrated that STRO-1^+^/c-Kit^+^/CD34^−^ hDPSCs show either a higher proliferation capacity and a lower doubling time, compared to STRO-1^+^/c-Kit^+^/CD34^+^ hDPSCs; indeed the latter exhibited, as early as at day 5, a slowdown in proliferation kinetics and, contrary to STRO-1^+^/c-Kit^+^/CD34^−^ hDPSCs, showed a reduction in cell density over the culture time. Further analyses highlighted an appreciable cell suffering in STRO-1^+^/c-Kit^+^/CD34^+^ hDPSCs, which resulted early induced towards cell senescence, as revealed by the high number of cells positively labeled through the β-galactosidase assay; moreover cell apoptosis could be detected as early as at passage 6, another event that was not observed in STRO-1^+^/c-Kit^+^/CD34^−^ hDPSCs subpopulation. Still at passage 6, the expression of the stem cell markers used to immune-select the two subpopulations was evaluated, showing that STRO-1^+^/c-Kit^+^/CD34^−^ hDPSCs were actively proliferating and maintained the expression of those stemness markers. Conversely, in STRO-1^+^/c-Kit^+^/CD34^+^ hDPSCs, the expression of CD34 was completely lost, while STRO-1 and c-Kit expression was maintained.

These data are in accordance with findings reviewed by Lin et al. [61] showing that after *in vitro* expansion and cell culturing a loss of CD34 expression was observed also in bone marrow mesenchymal stem cells [62]. Moreover, the culture conditions themselves might be the basis of the differences highlighted in CPD and PDT values and of the loss of CD34 expression. Although the function of CD34 surface marker is still unknown, it has been related to facilitation or inhibition of adhesion, cell proliferation and regulation of differentiation [63,64]. Particularly, as reported by Sidney et al. [65], cell culture on plastic surface in serum-containing medium could create an environment dissimilar to the in vivo environment, forcing the cells towards a progressive arrest of proliferation and senescence.

Relevant data regarding these two subpopulations were also provided by the evaluation of their differentiation potential *in vitro*, following the use of equal culture conditions.

No significant differences have emerged from the induction towards the osteogenic, adipogenic and myogenic lineages, in fact, the two subpopulations showed similar abilities in the expression of lineage specific markers. With regard to the osteogenic differentiation, both STRO-1^+^/c-Kit^+^/CD34^−^ hDPSCs and STRO-1^+^/c-Kit^+^/CD34^+^ hDPSCs, after the induction, showed the expression of bone related proteins, such as osteocalcin, osteopontin, osterix and type I collagen, besides an active deposition of mineralized extracellular matrix.

The induction towards the adipogenic commitment produced the formation of lipid rich vacuoles by both the two subpopulations.

After the direct co-culture and differentiation together with C2C12 mouse myoblasts either STRO-1^+^/c-Kit^+^/CD34^−^ hDPSCs and STRO-1^+^/c-Kit^+^/CD34^+^ hDPSCs demonstrated the capability to actively participate in new mature myotubes formation; in particular, the newly formed myotubes were clearly hybrid and therefore derived by the fusion of human stem cells with mouse myoblasts, as confirmed by the positive staining with antibodies specific for mitochondrial and human proteins, respectively. In particular, the newly formed myotubes were hybrid and, therefore, derived by the fusion of both human stem cells and mouse myoblasts, as confirmed by the labeling with antibodies specific for human mitochondrial protein and human nuclei, respectively.

A notable difference between the two subpopulations emerged from the evaluation of the induction towards the neurogenic commitment. Interestingly, STRO-1^+^/c-Kit^+^/CD34^−^ hDPSCs showed a much lower efficiency of commitment compared to STRO-1^+^/c-Kit^+^/CD34^+^ hDPSCs, as demonstrated by β-III tubulin expression (2% vs 85% positive cells, respectively) and by the shift to a neuronal-like shape, following the induction.

In particular, STRO-1^+^/c-Kit^+^/CD34^+^ hDPSCs also showed the expression of further markers, such as MAP-2, Neu-N and synapsin, confirming a substantial commitment towards neuronal lineage. The neurogenic induction was also able to induce the differentiation towards glial-like cells, as shown by the staining against GFAP. Overall, the analysis of these two subpopulations would suggest that a different embryological derivation exists, which would allow to distinguish the STRO-1^+^/c-Kit^+^/CD34^−^ hDPSCs, derived from mesoderm layer, and the STRO-1^+^/c-Kit^+^/CD34^+^ hDPSCs, whose origin might be traced back to the ecto-mesoderm and therefore due to neural crest cells migrating during the embryo development.

In support of this hypothesis, the expression of two specific markers, nestin and CD271, was evaluated in both STRO-1^+^/c-Kit^+^/CD34^−^ hDPSCs and STRO-1^+^/c-Kit^+^/CD34^+^ hDPSCs.

Nestin, a component of the intermediate filament proteins which form the cytoskeleton, is mostly expressed by neuronal stem cells [66]; the surface antigen CD271 is one of the two receptors for neurotrophins, growth factors that promote neuronal cells survival, besides being a marker that identifies neural crest derived cells.

The evaluation of the expression of these two markers in both STRO-1^+^/c-Kit^+^/CD34^−^ hDPSCs and STRO-1^+^/c-Kit^+^/CD34^+^ hDPSCs after standard culture conditions, without any induction towards the differentiation, revealed that STRO-1^+^/c-Kit^+^/CD34^−^ hDPSCs did not show any expression of nestin and CD271, whereas the STRO-1^+^/c-Kit^+^/CD34^+^ hDPSCs demonstrated a strong expression of both the markers.

These further data support findings from Laino and colleagues who formerly demonstrated that combined expression of STRO-1, c-Kit and CD34 by hDPSCs allowed to isolate a subpopulation of mesenchymal stem cells of neural crest origin [42]. Notably, as reported in literature, a few neural crest cells are known to be pluripotent, with self-renewal ability following migration, which are features of stem cells, thus termed “neural crest stem cells” (NCSCs). NCSCs have been isolated from different adult tissues and can differentiate towards mesenchymal lineage and neural lineage *in vitro*. It is noteworthy that our latter results are in accordance with findings from Abe and colleagues, reporting that sphere-forming cells derived from stem cells of apical pulp tissue express either NCSCs marker CD271 and neuronal stem cell marker nestin, and are also able to undergo neuronal differentiation [33].

Farther, in a previous study by Lizier and colleagues, dental pulp was demonstrated to have multiple stem cell niches, which are localized in capillaries and nerve networks in “cell free zone”, within the innermost pulp layer in “cell rich zone”, and in the outermost layer containing odontoblasts, being all these niches rich of nestin positive cells [67]. According to previous findings it is remarkable that STRO-1 antigen also recognizes a small fraction of CD34^+^ stem cells [37,20], and that is localized in large blood vessels of different adult tissues [35], including dental pulp [57]. In light of these considerations, our findings allow to infer that STRO-1^+^/c-Kit^+^/CD34^+^ hDPSCs reside in a perivascular niche, besides representing a stem cell population of neural crest origin.

The results obtained in this study highlight the heterogeneity of the stem population residing within the human dental pulp, particularly, its peculiar embryological origin, might explain the existence of two distinct subpopulations.

## Conclusions

The results obtained in our study are consistent with previous reports from Laino et al. [20,42], in which DPSCs positively sorted for the markers STRO-1, c-Kit and CD34, were shown to be capable to differentiate towards osteogenic, adipogenic and myogenic lineages, although they can be also differentiated toward further cytotypes. For these reasons, we have used the simultaneous expression of CD34, STRO-1 and c-Kit to isolate a population of mesenchymal stem cells of neural crest origin, thus confirming that neural crest-derived stem cells are also present within the adult body, entrapped within dental pulp [9]. In conclusion, although these results are promising, further investigations are required in order to define the flexibility of application of hDPSCs in regenerative medicine. Moreover, to fully characterize a population of stem cells it is most likely that a specific marker profile would be required, alongside differentiation assays and functional profiling.

Likewise, if culture and propagation techniques for STRO-1^+^/c-Kit^+^/CD34^+^ hDPSCs can be optimized in order to obtain a sufficient number of cells combined to a reduction of PDT, these cells might represent a source of progenitor cells that can be exploited clinically in regenerative medicine strategies.

### Ethical approval

No ethical approval by University Research Ethics Committee was necessary for this study.

## Abbreviations

hDPSCs: Human dental pulp stem cells
hAFSCs: Human amniotic fluid stem cells
SHEDs: Stem cells from human exfoliated deciduous teeth
SCAPs: Stem cells from apical papilla
PDLSCs: Periodontal ligament stem cells
DFPCs: Dental follicle progenitor cells
MSC: Mesenchymal stem cell
SCF: Stem cell factor
HSC: Hematopoietic stem cells
BM-MSCs: Bone marrow mesenchymal stem cells
ADSC: Adipose derived stem cells
FCS: Foetal calf serum
MACS: Magnetic-activated cell sorting
Abs: Antibodies
SD: Standard deviation
PDT: Population doubling time
PD: Population doubling
CPD: Cumulative population doubling
PBS: Phosphate buffer saline
OPN: Osteopontin
Osx: Osterix
OCN: Osteocalcin
hMit: Human mitochondria
hNu: Human Nuclei
Coll-I: Type I collagen
RFU: Relative fluorescent unit
NCSCs: Neural crest stem cells
MAP2: Microtubule-associated protein 2
GFAP: Glial fibrillary acidic protein
